# Periosteal Sharpey’s fibers: a novel bone matrix regulatory system?

**DOI:** 10.3389/fendo.2012.00098

**Published:** 2012-08-09

**Authors:** Jean E. Aaron

**Affiliations:** Bone Structural Biology Laboratory, Faculty of Biological Sciences, University of Leeds, Leeds, Yorkshire, UK

**Keywords:** collagen type III, collagen type VI, tenascin, elastin, matrix biochemical domains, skeletal aging, endosteal membrane

## Abstract

Sharpey’s “perforating” fibers (SF) are well known skeletally in tooth anchorage. Elsewhere they provide anchorage for the periosteum and are less well documented. Immunohistochemistry has transformed their potential significance by identifying their collagen type III (CIII) content and enabling their mapping in domains as permeating arrays of fibers (5–25 μ thick), protected from osteoclastic resorption by their poor mineralization. As periosteal extensions they are crucial in early skeletal development and central to intramembranous bone healing, providing unique microanatomical avenues for musculoskeletal exchange, their composition (e.g., collagen type VI, elastin, tenascin) combined with a multiaxial pattern of insertion suggesting a role more complex than attachment alone would justify. A proportion permeate the cortex to the endosteum (and beyond), fusing into a CIII-rich osteoid layer (<2 μ thick) encompassing all resting surfaces, and with which they apparently integrate into a PERIOSTEAL-SHARPEY FIBER-ENDOSTEUM (PSE) structural continuum. This intraosseous system behaves in favor of bone loss or gain depending upon extraneous stimuli (i.e., like Frost’s hypothetical “mechanostat”). Thus, the birefringent fibers are sensitive to humoral factors (e.g., estrogen causes retraction, rat femur model), physical activity (e.g., running causes expansion, rat model), aging (e.g., causes fragmentation, pig mandible model), and pathology (e.g., atrophied in osteoporosis, hypertrophied in osteoarthritis, human proximal femur), and with encroaching mineral particles hardening the usually soft parts. In this way the unobtrusive periosteal SF network may regulate bone status, perhaps even contributing to predictable “hotspots” of trabecular disconnection, particularly at sites of tension prone to fatigue, and with the network deteriorating significantly before bone matrix loss.

## PERIOSTEUM AND SHARPEY’S FIBERS

### PERIOSTEUM

This strong, encapsulating skeletal membrane containing osteoprogenitor cells consists of an outer fibrillar layer and an inner cellular layer that is usually poorly defined unless actively engaged in osteoid apposition. Despite its relatively low visual impact it defines vital developmental boundaries. Extending from it are the Sharpey’s fibers that ensure adhesion to the outer cortex and to tendons and ligaments, themselves perceived as modified periosteum (Hurle et al., 1989). While the unstressed periosteum seems biochemically quiescent, short bursts of loading stimulates the rapid induction of enzyme activity within discrete periosteal and bone matrix domains (Skerry et al., 1989) apparently by the mediation of signals to selected regions.

### SHARPEY’S FIBERS

**FIGURE 1:**
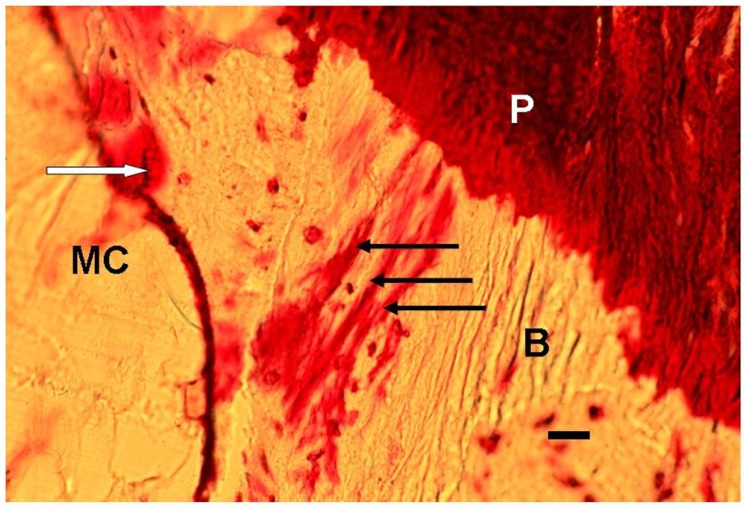
**Photomicrograph of a typical representative array of three periosteal Sharpey’s fibers (black arrows), each about 15 μm thick, and extending from the periosteum (P), through the bone (B) toward the endosteum outside which is the marrow cavity (MC).** In addition, nearby are stress-induced microcracks, for example, one (white arrow) is surrounded by bone matrix which has become “leaky” to the stain (normally impervious) apparently due to submicroscopic fatigue fissures increasing accessibility of the dye. Human proximal femur. *En bloc* gentian violet stain. Scale bar 30 μm.

**FIGURE 2:**
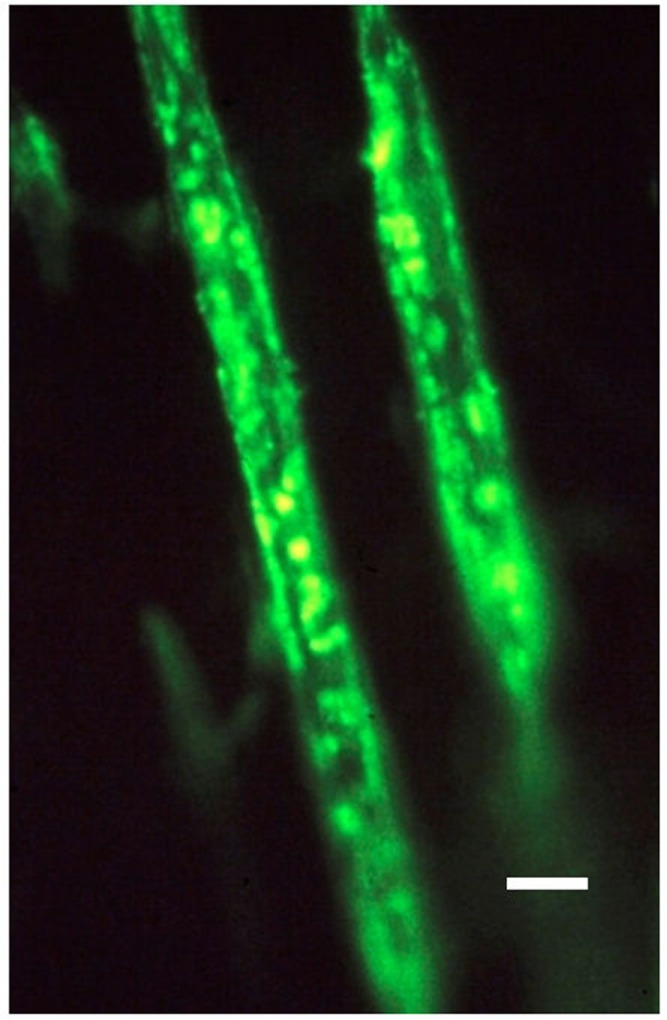
**Photomicrograph of two typical representative collagen type III-rich Sharpey’s fibers, about 10 μm thick, and fluorescing positive within the negative calcified bone matrix.** Human proximal femur. FITC-immunostain for CIII, UV epifluorescence microscopy. Scale bar 10 μm. After Carter et al. (1992).

These delicate optical features (**Figures 1 and 2**) described as “perforating fibers” by William Sharpey, cross matrix lamellae and are particularly abundant in the alveolar socket of the teeth (Sharpey et al., 1867). Also reporting them at this time was H. Muller (*Quain’s Elements of Anatomy*, 1867) who recognized the elastic nature of the fibers and a tendency to “escape calcification.” Later Weidenreich (1923), citing Koelliker (1886), confirmed their poorly mineralized status, and although they were apparently short and superficial he was of the opinion that they influenced not only the external anatomy but also the internal bone structure. From another quarter were reports by Tomes (1876) and Black (1887) that embedded Sharpey’s fibers constituted the cemento-alveolar fibers of the periodontal ligament, and in due course Cohn (1972) mapped their passage through the cementum and on across the entire thickness of the alveolar wall (Quigley, 1970). Other related reports followed, such as that by Jones and Boyde (1974) outlining further their presence in the cranial sutures and muscle attachments as well as in tooth sockets. However, the subsequent literature focused almost exclusively on Sharpey’s fibers functioning as the periodontal ligament and how this special dental structure altered with age both organically and inorganically, weakening its tooth-holding capacity. The detrimental changes observed included fibrosis, increased cellularity, and progressive calcification (Sloan et al., 1993).

At the present time, sufficient evidence is now accumulating to suggest that the relative neglect of those abundant Sharpey’s fibers located away from the dentition may be unjustified. In redressing the balance in favor of their structural significance elsewhere in the skeleton, and complementing the classification of Johnson (1987), Al-Qtaitat (2004), 2007 identified two types of Sharpey’s fibers (see also Al-Qtaitat et al., 2010), one coarse (8–25 μm thick) and the other fine (<8 μm thick). Their entry angle into the subperiosteal bone was multiaxial. It included the almost horizontal (i.e., tangential) fibers especially common with age and often found among inserting muscle fascicles, functionally propagating biomechanical exchange across the periosteum. It also included the perpendicular (i.e., vertical) fibers, frequently crossing the cortex to the cancellous region and generally of the coarse type in bundles <40 μm thick, functionally adding complexity to the muscle-to-bone interface that may influence bone atrophy, augmentation, and remodeling. In addition were the oblique fibers, these being the most numerous and predominant in the young skeleton, functionally mediating exchange between the periosteum and outer cortex and providing soft tissue anchorage. While some of these insertions apparently ended abruptly (like rows of short, regular parallel stitches), it was the proportion that traversed to the medulla, some becoming intertrabecular, others with dispersed intra-osseous fan-like termini that were of special interest. Added to this was their unusual profile in transverse section, which was not the simple circle expected but showed sharply defined surface indentations and configurations ranging from a horseshoe-shape to a “hollow” core (Aaron and Skerry, 1994).

Further examination using an established histochemistry test for elastin (Verhoeff’s stain) supported the observation of Muller above that (unlike collagen type I, CI) they have elastic properties that can absorb strain. Moreover, the elastin staining was not uniform but suggested the discrete contours of a spiral encircling some of the individual coarse fibers (Aaron and Skerry, 1994). The mechanical properties of elastin are unique. Unlike non-extensible collagen it can be stretched, recoils, branches, and imparts flexibility. However, it has been rarely documented in bone (Johnson and Low, 1981; Keene et al., 1991), except, that is, at sites of tendon and ligament insertion, and its presence will alter the biophysical properties of the Sharpey’s fibers.

### IMMUNOHISTOCHEMISTRY OF SHARPEY’S FIBERS

It required a technological advance to demonstrate the otherwise hidden scale of Sharpey fiber permeation and to establish their biochemical composition more extensively (see Aaron and Shore, 2004b for technical details). Polarized light showed a highly birefringent nature consistent with collagen, but little else could be deduced by simple staining (Smith, 1960), with for example picro-sirius red stain, or by the Goldner tetrachrome method (Aaron and Shore, 2004b). The prospect was transformed by the introduction of heavy duty cryomicrotomy (see for example, Aaron and Carter, 1987; Carter et al., 1989), combined with the increasing availability of a widening range of specific fluorescent antibodies. Prior to this, the organic matrix biochemistry was based on tissue homogenates and extracts. The new method enabled a structural face to be applied. This identified previously unsuspected matrix sub-divisions, showing a mosaic of biochemically distinct domains, defined by boundaries and with differential aptitudes for signal trafficking through, for example, endochondrally derived versus intramembranous regions. Perhaps foremost among these potentially transducing macomolecules is collagen type III (CIII). It is this together with amounts of collagen type VI (CVI), tenasin, fibronection, and elastin, that are now known to characterize the Sharpey’s fibers, meaning that structurally they are considerably more complex than was previously supposed, and especially complex for structures fulfilling the relatively uncomplicated function of anchorage traditionally assigned to them.

An advance in their histochemical differentiation had pre-empted their immunohistochemistry with descriptions of certain “argyrophilic” matrix fibers (Nowack et al., 1976; Carter et al., 1991 for references), which in retrospect were found to be coincident in distribution with CIII immunostaining. As with the more prominent CI, so CIII is also found in all interstitial connective tissues but in contrast there was little evidence for its occurrence in bone, with the exception of the earliest mesenchymal condensations (Pratt, 1959; Page et al., 1986), and its well documented appearance in alveolar bone (Becker et al., 1986). The application of CIII immunostaining (**Figure 2**) has now transformed this state of affairs (Wang et al., 1980), leaving in no doubt its discrete structural affinity for the birefringent Sharpey’s fibers.

#### Collagen type III

It is recognized that different collagens, e.g., CI and CIII can be present in the same fibril to modulate its physical properties. Like CI, the structure of CIII consists of long (300 nm) uninterrupted triple helices, chemically distinguished from CI by an increased level of 4-Hyp and the occurrence of cysteine, facilitating disulphide bond formation. In contrast to the high tensile strength of CI fibers, those of CIII are thinner and less orderly (Kielty et al., 1993) and they are prevalent in tissues with clear elastic properties, including skin, aorta, lung, and gut. As well as being argyrophilic, above, these fibers were known histologically as reticulin fibers, and were especially associated with epithelial basement membrane stability, where their contribution to organ containment cannot be overestimated. It was reported by Bailey et al. (1993) that in normal human bone CIII content averaged 4–5%, with 3% in osteoporotic bone. Similarly in culture conditions osteoblast-like cells have been said to secrete about 6% CIII (Aufmokolk et al., 1985; see Luther, 1998 for references). The collagens CI, II, and III are all translated from mRNA coding for pre-proα chains with similar, but not identical, N- and C-terminal extensions. The partnership of CIII with CVI is reported to provide exceptional stability (e.g., Hulmes, 1992; Sherwin et al., 1999) and this combination in Sharpey’s fibers must have fundamental implications for their persistence in a tissue with the versatility and turnover of bone.

#### Collagen type VI

A structural association between CIII and CVI in bone was reported by Becker et al. (1986), and CVI was said to be reduced in osteoporosis (OP; Bailey et al., 1993) although the implications were not clear. CVI is microfibrillary, composed of a short triple helical axis and globular termini, creating its typical dumbell shape. It has many adhesive RGD sequences and like CIII has stabilizing disulfide bonds (Hulmes, 1992). It has been suggested that the removal of CVI is a factor that may permit remodeling (Sloan et al., 1993).

**FIGURE 3:**
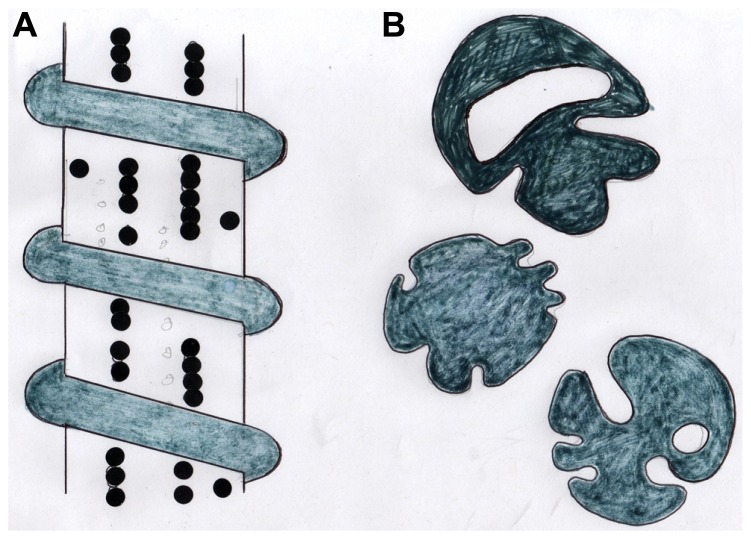
Diagram showing **(A)** a stylized CIII/CVI-rich periosteal Sharpey’s fiber with adherent beaded chains of tenascin and encircled by a coil of elastin, and **(B)** tracings of the same coarse fibers (about 15 μm diameter) in cross section showing their typical irregular profiles. After Aaron and Skerry (1994).

From the above evidence it is clear that Sharpey’s fibers are uniquely placed and have the morphological complexity (**Figure 3**) to mediate musculoskeletal cohesion and exchange. They are the only continuous anatomical structure to (i) integrate directly with the muscles, ligaments, and tendons, (ii) traverse the periosteum from which they arise, and (iii) permeate the extracellular matrix multiaxially and to varying degrees. Insight into their most basic structural modulation may be found in tooth movement where orthodontic forces strengthen the CIII periodontal attachment (Wang et al., 1980) by increasing the diameter of the Sharpey’s fibers. Again, in calvarial bone, the Sharpey’s fibers are organized relative to the pull of the masticatory muscles (Simmons et al., 1993), while in spaceflight there is apparently disorganization of the subperiosteal collagen fibrils (Wronski and Morey, 1983; Vailas et al., 1988).

There now follows seven reasons why the Sharpey’s fiber network may act as an extracellular regulatory system in bone. Its candidature has been a lengthy one. Though not assigned as such, elements of the trabecular framework proposed below probably commenced in the seventeenth century at the dawn of microscopy with descriptions by Clopton Havers (Dobson, 1952) of penetrating “fibrillae,” thereby possibly preceding Sharpey himself. The precise nature of the musculoskeletal exchange mechanism instigated remains to be established, for example, a piezoelectric phenomenon (the piezoelectric modulus of tendon is apparently 30-fold that of bone; Marino and Becker, 1971) or one involving stress-regulated excitatory amino acids analogous to neural pathways (Mason et al., 1997) may be considered; there is also evidence that the ligaments with which the Sharpey’s fibers integrate may function as proprioceptors (Johansson et al., 1991).

### SHARPEY’S FIBERS IN FETAL BONE DEVELOPMENT (FIBRONECTIN AND TENASCIN FACTORS)

A system of Sharpey’s fibers continuous with the ectodermal membrane is present from an early embryonic stage. They appear as dorso-ventral fibrillar bundles, about 1μm thick, containing also CI, fibronectin, and tenascin. They occupy an area that becomes an intracortical CIII-rich domain in the limb bud that is linked to tendon generation (Hurle et al., 1989) and variations from the norm can have pathological consequences. This is illustrated by comparing intramembranous bone development in the normal human femoral anlagen with that of dysplastic lesions (Carter et al., 1991). Key structural molecules in the genesis of new trabeculae are not only collagen types III and VI, but also adherent are the glycoproteins tenascin and fibronectin. Regarded as “biological organizer” molecules they carry the adhesive RGD sequence, fibronectin apparently influencing fibroblast migration. However, in relation to Sharpey’s fibers it is tenascin that seems to have a special role, where it may mediate attachment of osteoblasts by means of its cell recognition signal (Ruoslahti and Pierschbacher, 1986). The occasional surface location of alkaline phosphatase on some fibers may relate to this signal and may indicate the expansion of thinner fibers with circumferential apposition in response to brief loading (Aaron, 1980b). Immunostaining for tenascin indicates that it adopts a highly characteristic beaded pattern (**Figure 3**) the linear alignment of which is critical for normal development, as follows.

Contiguous with the periosteum surrounding developing intramembranous bone are arrays of CIII-rich Sharpey’s fibers which apparently form a scaffold upon which the new trabeculae are assembled and the bone modeling event takes place. The framework is recognized by antibodies to CI and fibronectin, but these affinities disappear as the Sharpey’s fibers become surrounded by calcified bony tissue. Remaining in association, however, is tenascin in a remarkable regular beaded arrangement. The intramembranous bone formation can only apparently continue in an orderly manner toward maturity on condition that tenascin is specifically associated with the Sharpey’s fibers at this crucial stage. In its absence the bony tissue is permanently destined to remain disorganized and immature, as is the case in fibrous dysplasia (Sloan et al., 1989; Carter et al., 1991).

The preliminary framework appears to persist to maturity (being absent from endochondrally derived bone) as periosteal myotendinous insertions of Sharpey’s fibers. By providing this continuous, elastic (Keene et al., 1991) intermediary between the developing musculature and the developing bone matrix the CIII fibers may enable the translation of stresses generated by contractile tissues into compliant modeling and remodeling of the contiguous trabecular architecture in the femoral anlagen (Pratt, 1959; Wong and Carter, 1990). It may be envisaged that an understanding of such interactions between organizing proteins (like tenascin and fibronectin) and extracellular structures like CIII fibers which are fundamental to early trabecular development in the first stages of life may direct novel strategies for restitution of the atrophied skeleton in later life.

### SHARPEY’S FIBERS IN MATURE BONE REPAIR FOLLOWING ABLATION (THE ENDOSTEUM FACTOR)

Just as damage to the adult periosteum stimulates the polarized extension of its Sharpey’s fibers to re-establish lost continuity, so also does the endosteum appear to be similarly stimulated when damaged experimentally, as in the course of tissue ablation of a cylindrical hole in the ovine pelvic girdle caused by the removal of an 8 mm diameter trephine bone biopsy (Aaron and Skerry, 1994). Picking up the damaged threads, and considerably more numerous where there are bone fragments (a likely source of local growth factors), there arises from the excised surfaces marshaled arrays of uncalcified, discrete coarse (5–25 μm) birefringent fibers, converging centripetally. It is upon this assembly that the replacement primary trabeculae gain support, and in regions where the scaffold is absent, so also absent is trabecular genesis. This endosteally derived fibrous framework remains unmineralized and therefore apparently protected from osteoclastic resorption (Aaron, 1980a), aided by other inhibitory intrinsic factors such as CVI. It apparently survives, even when the thickening primary bars are significantly opened up by resorption channels into a typical network of mature secondary trabeculae. The outcome of this endosteal activity is the guaranteed presence of a persistent fibrillar assembly that crosses domain boundaries without interruption, bonding soft to hard tissues and new bone to old, and which seems central to a self-repair process of admirable efficacy. Thus, the subperiosteal trabecular generation of embryonic skeletal development in Section “Sharpey’s Fibers in Fetal Bone Development (Fibronectin and Tenascin Factors)” above is conserved and recapitulated subendosteally in the adult in response to insult (and possibly also sclerotic pathology such as Paget’s disease).

### SHARPEY’S FIBERS IN MATURE BONE AFTER OVARIECTOMY (THE HUMORAL SENSITIVITY FACTOR AND SYSTEM ATROPHY)

**FIGURE 4:**
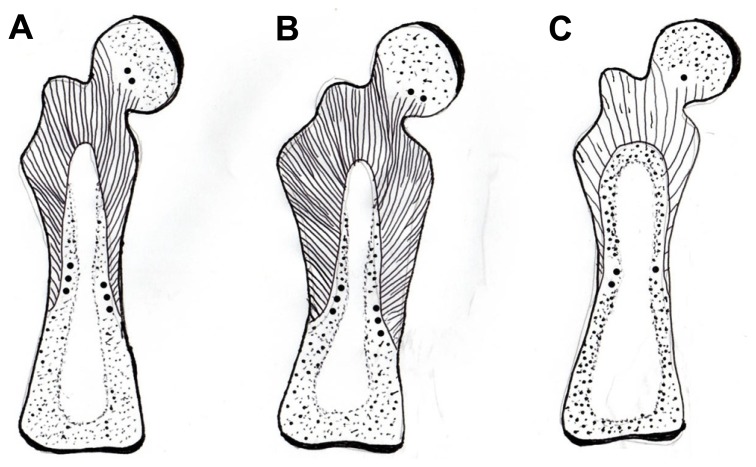
Diagram of a mature rat femur showing the gross configuration of the expansive CIII/CVI-rich proximal domain of Sharpey fiber bone (striped area), terminating in CII/CVI-rich “anchors” (round dots), **(A)** in a normal control, **(B)** expanded in response to voluntary running exercise, and **(C)** contracted in response to OVX. After Luther et al. (2003) and Saino et al. (2003).

The ovariectomized rat is an established model of bone loss precipitated by declining estrogen levels. In this model, the Sharpey’s fibers of the femur are especially evident and they are mainly confined within the boundaries of an extensive proximal domain which they serve to characterize. The configuration of this domain is such that three-dimensional maps are essential in its recognition (Luther, 1998; Luther et al., 2003), reliance on single thin histological sections revealing only the tip of the morphological iceberg. Resembling in transverse section a fluorescent subperiosteal collar after immunostaining for CIII, the domain commences about one-third along the shaft from the knee where two opposing subperiosteal stained sectors progressively expand and merge into a single circumferential band, which in turn broadens to occupy the entire width of the cortex at the level of the third trochanter and above. By other means Pratt (1959) had described a similar histological feature in the developing rat femur as “the primitive bony diaphysis” and earlier Weidenreich (1930) in a matching region in the adult human proximal femur reported a propensity of poorly calcified fibers associated with tendon insertions. The arrangement is so striking that the domain resembles a bony encapsulated tendon, well placed to maximize direct functional adaptation (**Figure 4A**). In the healthy animal the persistent character of Sharpey’s fibers again means that the region is uniquely resistant to remodeling. However, if estrogen levels fall, as is the case after ovariectomy (OVX), this stability apparently soon diminishes (Luther et al., 2003). This sudden change is expressed in the marked decline in immunostaining of the Sharpey’s fibers for CIII and VI and their disappearance from regions they had previously so clearly occupied (**Figure 4C**), an event that notably preceded any bone loss and cortical thinning that took place subsequently. Related to the negative change in the Sharpey’s fibers following OVX may be the process of muscle atrophy described at menopause (Burr, 1997), with an associated decline in muscle insertions. The apparent demise of the proximal domain in this way was accompanied by a significant reduction in both the length and the number of CIII/CVI fibers.

Insight into a remarkable example of form-and-function adaptation by the periosteum and its appendages came about unexpectedly in mapping the perimeter of the proximal domain above. Along the part of its inner boundary that occurs approximately mid-cortex were distributed at intervals, like small buttons, islands of cartilage, looking at first sight like remnants of endochondral ossification (**Figure 4**). However, it seems instead that they are discrete satellite patches of “trapped” periosteum (Zawisch-Ossenitz, 1927, cited by Luther, 1998) and were similarly observed by Pratt (1959). This explains their staining for CVI with its concentrated adhesive groups, as well as the more usual CII of cartilage. An association of these unusual objects with tendinous insertions is now recognized by a number of other authors (Benjamin and Ralphs, 1997; Clarke and Stechschulte, 1998; Benjamin et al., 2006), including Ellerstrom et al. (1998) who described them in growing rat alveolar bone as “flexible hinges.” As the name implies, they bring to the boundary of the mechanically and humorally responsive CIII-rich domain the essential capacity for differential adjustment, since it is an essential property of cartilage that it can expand intrinsically, unlike bone which cannot.

### SHARPEY’S FIBERS IN MATURE BONE AFTER EXERCISE (VOLUNTARY RUNNING; THE PHYSICAL SENSITIVITY FACTOR AND SYSTEM AUGMENTATION)

The rodent femoral proximal domain above is responsive to levels of physical activity and this has been illustrated by comparing its features in animals with voluntary access to a running wheel and those confined to their cage. Running increased subperiosteal apposition to a peak adaptation after which there was no more augmentation induced by prolonging the exercise period further. It was observed that the CIII-rich subperiosteal domain was almost 60% wider in the runners (**Figure 4B**), with a 15% rise in numerical density of the Sharpey’s fibers and a 7% increase in their thickness (Saino et al., 2003). The CIII fibers were densest near the proximal (i.e., hip) joint in contrast to their paucity in relation to the distal (i.e., knee) joint, suggesting that the anatomical avenue for musculoskeletal exchange was more limited in the lower region. This structural dichotomy relative to a possible proactive role in bone status of the fibers is supported by the independent observation of Newhall et al. (1991) that in rats only the bone mass of the proximal femur was responsive to running activity while the distal femur was inexplicably unchanged. It also follows that the highest levels of tension and compression occur proximally and decline distally (Rybicki et al., 1972).

From another quarter was a second independent piece of supporting evidence using young rabbits. Hert et al. (1972) mechanically loaded the tibia and found that the process stimulated intra-cortical haversian remodeling which did not seem unusual. Inexplicably, however, there was again a dichotomy in the response of the tissue. Only bone at the inner cortex that was derived endochondrally (i.e., devoid of Sharpey’s fibers) remodeled, while the overtly similar outer cortical bone that was derived intramembranously (i.e., populated by Sharpey’s fibers and where microstrain is at its highest near the perimeter) did not remodel. In practical terms this distinction between endochondral and intramembranous bone may be influential in the success rate when used surgically as tissue grafts (Wong and Carter, 1990).

**FIGURE 5:**
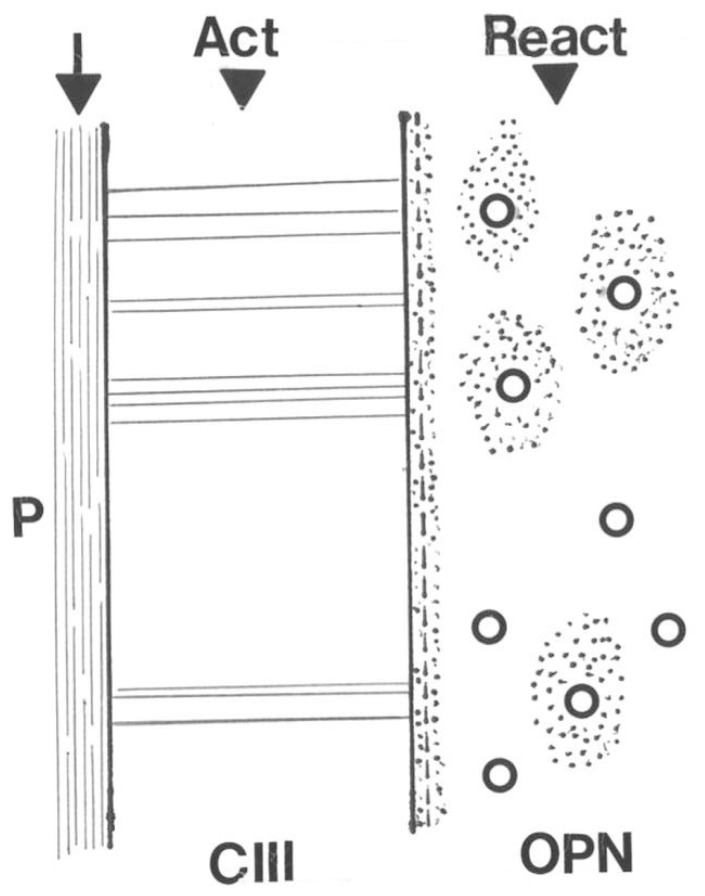
**Diagram illustrating the conductance of a hypothetical stimulus to the periosteal membrane (P) across the adjacent CIII-rich Sharpey fiber domain to its direct interface with the inner domain where the act is translated into a reaction of increased vascularity via osteopontin (OPN)-rich new haversian systems**.

Skeletal adaptation has been related to changes in bone blood flow (Weinbaum et al., 1994; Qin et al., 2001) and Sharpey’s fibers may be influential here too, this time indirectly. As suggested above, the outer CIII-rich domain is concentric to an inner domain from which CIII is entirely absent, except for a continuous band of CIII defining the discrete boundary between the two. Present also at the boundary is the non-collagenous protein osteopontin (OPN) as a thin immunofluorescent band that does not encroach upon the CIII-dominant domain. Nowhere else than here do the two coexist (i.e., they are apparently mutually exclusive). Instead, OPN enriches the matrix of the inner domain only (**Figure 5**), where it is not random but characterizes the appearance of new haversian systems and an associated vascularity which is significantly increased in the running rats. In other words, the two concentric domains differ in their immunohistochemistry and although they respond in unison, they react in histologically different ways to the same stimulus. An interpretation may be that the responsive CIII-rich subperiosteal domain detects the extraneous signal, responds and conducts it to its inner border, on the other side of which the inner domain enables its intrinsic translation (**Figure 5**). Thus, the voluntary exercise regime enhances the molecular distinction between the two concentric domains making the CIII-rich domain richer in CIII and the OPN-rich domain richer in OPN (**Figure 5**).

### SHARPEY’S FIBERS IN MATURE BONE WITH AGING (THE CALCIFICATION FACTOR)

As an intermediary between soft muscle and hard bone, the periosteum is as susceptible to the passage of time as are they. CIII is abundant in embryonic skeletal tissue development. With age, fibroblasts tend to synthesize more CI and less CIII, such that the general proportion of CIII is significantly less than in youth. Added to this, increased collagenase production has been attributed to fibrocytes under some conditions and may cause fiber degradation with time. In this way the periosteum and its Sharpey fiber appendage may change organically with age, and it may also change inorganically with implications both for musculoskeletal exchange and for bone structural “quality.”

Taking as a model the porcine mandible (because of its exceptionally powerful musculoskeletal bonding) it was observed by Al-Qtaitat et al. (2010; see also Al-Qtaitat, 2004 for references) that age-related changes in the periosteum included thinning and a diminution in the number, length, and birefringence of the associated Sharpey’s fibers. In addition, they became less ordered and less multiaxial, and instead were mainly superficial, horizontal, truncated insertions, with fewer of the transcortical and intertrabecular fibers of youth. While confocal microscopy confirmed the complexity of their immunofluorescent arrays and occasional fan-like termini, with aging the subperiosteal CIII-rich zone tended to fluoresce less brightly and narrowed, such that parts of the matrix previously positive were now negative (i.e., as for OVX above) and some cohorts of fibers seemed fragmented and disconnected.

**FIGURE 6:**
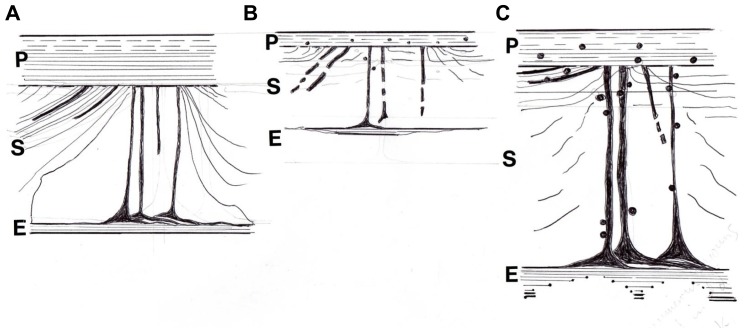
**Diagram of the postulated periosteal-Sharpey’s fiber-endosteal (PSE) integrated system**. **(A)** In youth when it is multiaxial and pervasive with oblique insertions predominant and with a well defined CIII-rich endosteal rim. **(B)** With age and osteoporosis when insertions are more polarized horizontally, with general signs of regression and fragmentation, and with a discontinuous CIII-rich endosteal rim; also associated are sparsely distributed fine mineral microparticles, about 1 μm diameter, that may alter flexibility. **(C)** In osteoarthritis, similarly characterized by more numerous horizontal insertions and a more heterogeneous incidence of prominent thickened fibers, and with a CIII-rich endosteal rim that is widened in places; also associated are frequent coarse mineral microparticles, about 2 μm diameter, that may increase stiffness. After Al-Qtaitat (2007) and Al-Qtaitat et al. (2010)).

As the periosteum reduced with time, it became more susceptible to calcification (**Figure 6**). At its most extreme this took the form of sporadic irregular dense aggregates, about 50 μm in diameter and showing little alignment with the fibers. However, more commonly observed were discrete, scattered calcified particles, about 1 μm in diameter, and resembling similar fine particles within the mineralized bone matrix itself (Aaron et al., 1999). These occurred separately and in linear chains within the periosteum or looped around some of the Sharpey’s fibers. Using a chromium labeling technique originally developed for mineral “ghosts” (Al-Qtaitat et al., 2010) it was indicated by EDX element microanalysis that the proportion of calcium, phosphate, and associated uptake of chromium label were all higher in the spectrum from the older pig than its young adult counterpart where counts were particularly low. While the looped mineral particulate chains in the older (though not frail) pig may increase structural strength and binding of insertions, as is the case in the spindle-legged turkey tendon, in excess they may compromise function. In their capacity as “micro-tendons” the calcification of pliant young Sharpey’s fibers may beneficially reduce excessive stress perception and buffer potentially damaging loads avoiding detachment of the periosteum and generally supporting a flexible structure in strategic places as needed. The larger deposits on the other hand, may cause unwelcome restriction and relate to calcifying tendonitis.

The age-related calcification of Sharpey’s fibers advances toward the periosteum which itself can be described as partly calcified and may cause functional incapacity. According to Jones and Boyde (1974) who wrote one of the few papers on this subject, the degree of mineralization of Sharpey’s fibers varies from a mineralized perimeter and unmineralized core, to calcification throughout. Thus, mineral microparticles transgress the discrete calcification front boundary of the hard tissues and invade the soft ones, to partially stiffen the periosteum and Sharpey’s fibers, thereby destabilizing them and exposing to resorption activity prime skeletal areas previously protected by their presence.

### SHARPEY’S FIBERS AND THE PENALTY FOR MATRIX STABILITY (THE MICROFISSURE FACTOR)

A price may have to be paid for the stability that Sharpey’s fibers impart to the regions in which they are prevalent. The cost is in terms of an increased susceptibility to fatigue microfissures, and is histologically analogous to the accumulation of fluoride in the matrix which inhibits its remodeling (Aaron et al., 1992). Fractures were an unexpected consequence due to the unchecked accumulation and propagation of microfissures which would otherwise have been remodeled and repaired. The incidence of microfissures is not easy to determine reliably as the preparatory slicing for microscopy can artificially produce them. Prior soaking of intact specimens in histological dyes, such as basic fuchsin, gentian violet, or tetracycline stain, as was originally proposed by Frost (Aaron and Shore, 2004a) to permeate the matrix cracks is one option. It was found by extrapolating from this that heavy metal-containing dyes, such as the von Kossa silver stain have the advantage of electron density, enabling at least the larger cracks to be labeled and captured by the less tissue-destructive technique of micrcomputed tomography (micro-CT; P. A. Shore and J. E. Aaron, unpublished result). Such procedures suggest that microfissures are not unusual within the immediate vicinity of Sharpey’s fibers (see for example the small crack near the Sharpey array in **Figure 1**, top left), perhaps caused by local fatigue due to nearby repetitive muscle, tendon or ligament pull inducing failure by matrix crystal fracture or mineral microparticle slip (Aaron, 2003). The eradication of the microcracks would risk weakening insertion anchorage by permitting osteoclast access into the normally closed domain. On the other hand, without recovery the compromised region may destroy itself by the local release of hydrolytic enzymes (i.e., “suicide bags”) such as acid phosphatase, in the disintegrative process of autoclasis (Aaron, 1977).

### SHARPEY’S FIBERS IN MATURE BONE (THE UNIFYING CIII RESTING OSTEOID FACTOR)

Because the adult human proximal femur is an exceptionally vulnerable site it was the location of choice of Al-Qtaitat (2007) in transposing some of the observations in animal models above to the human condition. He was able to confirm the presence of CIII-rich Sharpey’s fibers as regular features of the femoral head and neck. At the same time there was an unexpected turn in events with the preliminary indication of another facet to the periosteal–Sharpey fiber assembly in this region, albeit at first glance an unprepossessing one. This was in the form of a fine discrete CIII-immunofluorescent rim which apparently outlined much of the endosteal surface, including the spongiosa, and which during observation had to be separated from an interfering non-specific autofluorescence that typically occurred along the trabecular edges (Al-Qtaitat, 2007). For this reason it was tempting to dismiss it, but before doing so a possible explanation was sought. Metabolically inert (resting) surfaces comprise approximately 80% of the total bone surface, the remainder being occupied by osteoid borders and resorption cavities. Osteoid borders are normally about 10 μm thick, however, it has been recognized for some time that a much narrower osteoid layer, about 2 μm thick (i.e., no more than one or two lamellae wide), and confirmed ultrastructurally, enshrouds all surfaces, except resorption cavities (Vanderweil, 1980). Speculation about the purpose of this recurrent and extensive phenomenon included the possibility that it was a barrier preserving the marrow tissue from calcification. Another equally pivotal role was in protecting the calcified matrix beneath from random osteoclastic access, until associated “resting” osteoblasts received the signal to release their stored collagenase for its selective removal. The layer had formerly been described as superficial, uncalcified collagen on a band of acid mucopolysaccharides and mineralization inhibitory factors (Raina, 1972; Vanderweil, 1980). The recent preliminary observation identifying a CIII-rich rim suggests that this constitutes at least a proportion of its make-up.

With the emergence now of preliminary evidence for a CIII-rich endosteum, there was raised the possibility that the proposed fibrous regulatory system is not confined to the outer periosteum and its Sharpey’s fibers, but that it may include the inner endosteum as well. In this way the two apparently separate structural membranes are evidently conjoined by the Sharpey’s fibers into a poorly calcified, pervasive and cohesive system of bone matrix containment (**Figure 6A**), to which might be attributed a sensory capacity for musculoskeletal exchange. Age-related (or osteoblast-directed) calcification of the inner rim would remove its defence against resorption (Aaron, 1980a), tipping the remodeling balance toward trabecular disconnection and structural weakness. There is also the possibility that the integrated system is the histological program for Frost’s theoretical “mechanostat” (Frost, 1964), which determines whether bone should be added or removed to retain its constant optimal loading capacity that is specifically set for each region. I think the notion might have been well received by him and maybe, to use his term, he would even think it “neat.”

#### Application of the proposed “Periosteum–Sharpey’s Fiber–Endosteum System”

Fresh human material is not readily available for research purposes, however, surgical discards from the aging human proximal femur accumulate daily from fracture (OP) subjects and non-fracture (osteoarthritis, OA) subjects. A pilot study by Al-Qtaitat (2007) was performed on material from the Orthopaedic Department (courtesy of Dr L. D. Hordon and the Orthopaedic surgeons of Dewsbury District Hospital (Mid Yorkshire Hospitals NHS Trust, with ethical committee approval and informed patient consent). In both groups, CIII-rich Sharpey’s fibers were confirmed in the femoral neck region in numbers sufficient to contribute to the structural “quality” of this clinically significant site. Immunostaining combined with epifluorescent and confocal microscopy enabled the mapping of the subperiosteal extensions which were traced toward the fluorescent endosteum Al-Qtaitat, 2007) via cortical and cancellous regions (**Figure 6A**). Subjective appraisal of the two pathologies representative of bone at opposite ends of the histopathological spectrum suggested differences in the postulated periosteum – Sharpey’s fiber – endosteum (PSE) system. The atrophied tissue remaining in the OP neck contained discontinuous and fragmented vertical insertions from the periosteum (i.e., a tenuous PSE system) with the mineral in their vicinity of a finely granular character (**Figure 6B**). In contrast, the hypertrophied irregular tissue in the OA neck contained fiber-rich and fiber-poor areas with sporadic, heterogeneous but prominent insertions from the periosteum with continuous horizontal fibers especially numerous (i.e., a substantial PSE system) and with a coarsely granular mineral that permeated the periosteum, perhaps reducing its flexibility (**Figure 6C**). While these apparent differences require confirmation, they may begin to provide new insight into the two major intractable causes for age-related disability and their future treatment.

## CONCLUSION

Clearly there is much to do to support and verify the evidence presented above and the interpretation placed upon it of a novel, sensory regulatory system within the bone matrix. However, a number of possibilities are raised concerning populations of discrete, uncalcified CIII/VI-rich fibers of structural complexity in the periosteum, in the endosteum, and in varying numbers, along diverse axes, in the bone in between, Added to this is their partial inundation in certain circumstances, such as aging, with calcified microspheres (fine in OP, coarse in OA), which will modulate their properties and in moderation may serve to facilitate, divert or to dampen their function, only becoming clinically problematic in excess. In brief, there is reason to suppose that the Sharpey’s fibers:-

- alter the structural “quality” of the bone matrix they occupy.
- are instrumental in early musculoskeletal development.
- provide an integrated scaffold for skeletal self-repair.
- are protected from resorption when in an uncalcified state, creating stability.
- if they calcify at the endosteal rim (i.e., resting surface osteoid layer) lose their protected status, enabling remodeling access and a potential imbalance in favor of trabecular disconnection and instability.
- by creating stability where they predominate, predispose to unrepaired fatigue microfissures.
- are apparently a direct microanatomical link uniting the outer periosteal and inner endosteal membranes, thereby crucially coordinating bone behavior.
- may weaken and fragment in circumstances such as a low estrogen status, reducing musculoskeletal exchange below a threshold that predisposes to OP.
- may strengthen and augment in circumstances such as increased activity, raising multiaxial musculoskeletal exchange beneficially; it may become detrimental if exchange becomes irregular, uniaxial, and excessive predisposing to OA.
- may be the histological basis for the theoretical “mechanostat” of Frost by signaling to each versatile skeletal location how it should behave to retain its predetermined optimum loading capacity.

Finally, what can be concluded about the “chicken and egg” conundrum, i.e., which came first? The present evidence suggests that prior to bone matrix atrophy, either after OVX (rat femur model) or with aging (pig mandible model), sites that had previously been periosteal SF-positive become negative. On this histological basis these intraosseous components with their extraosseous origins do not seem to be responding *in concert* with the bone to some other extracellular signal, but rather are a factor directly pre-empting a *sequential* event. To summarize, as intramembranous bone volume accumulates within the boundary-defining periosteum upon a signal-sensitive Sharpey fiber scaffold, so in those places where the scaffold fails the extracellular matrix may be selectively destroyed as the stimuli essential for its maintenance and the *status quo* fail to be delivered. In this way the CIII-rich Sharpey’s fibers as periosteal/endosteal membrane intermediaries may be as essential to the regulation and retention of functioning bone within the musculature as they are to the retention of functioning teeth within the gums.

## Conflict of Interest Statement

The author declares that the research was conducted in the absence of any commercial or financial relationships that could be construed as a potential conflict of interest.
